# Clinical characteristics of pyogenic spondylitis and psoas abscess at a tertiary care hospital: a retrospective cohort study

**DOI:** 10.1186/s13018-018-1005-9

**Published:** 2018-11-28

**Authors:** Tsukasa Nakamura, Takeshi Morimoto, Kosuke Katsube, Yuji Yamamori, Junji Mashino, Kiyoshi Kikuchi

**Affiliations:** 1 Department of Infectious Diseases, Shimane Prefectural Central Hospital, Izumo, Japan; 2 Clinical Education and Research Center, Shimane Prefectural Central Hospital, Izumo, Japan; 3 Department of Clinical Epidemiology, Hyogo College of Medicine, Nishinomiya, Hyogo 663-8501 Japan; 4 Department of Orthopedics, Shimane Prefectural Central Hospital, Izumo, Japan; 5 Department of Emergency Medicine, Shimane Prefectural Central Hospital, Izumo, Japan; 6 Department of General Medicine, Shimane Prefectural Central Hospital, Izumo, Japan; 7 Department of Pediatrics, Shimane Prefectural Central Hospital, Izumo, Japan

**Keywords:** Pyogenic spondylitis, Psoas abscess, Mortality

## Abstract

**Background:**

Psoas abscess and pyogenic spondylitis are intractable diseases that require long-term treatment, but the clinical characteristics and causative organisms have not been fully investigated. Herein, we describe the clinical characteristics of these diseases and evaluate the factors associated with in-hospital mortality and the presence of gram-negative rods as causative microorganisms.

**Methods:**

All patients diagnosed with pyogenic spondylitis or psoas abscesses at a tertiary hospital were included. We retrieved the clinical data (age, sex, outcome, length of hospital stay, disease, bacteria, medication, comorbidities, and treatment status), vital signs (blood pressure, heart rate, and body temperature), and laboratory test results (blood cell count, liver function, renal function, electrolytes, blood sugar, and C-reactive protein) of all patients. The outcomes were in-hospital deaths and positive cultures of gram-negative rods.

**Results:**

We analyzed 126 patients consisting of 69 (55%) men with a population mean age of 72 years. Seventy-two patients had pyogenic spondylitis and 54 had psoas abscesses. Eleven patients (8.3%) died during admission. The causative bacteria were gram-positive cocci in 63 patients (50%) and gram-negative bacteria in 19 patients (15%). The multivariate logistic model showed that blood urea nitrogen (BUN) (odds ratio [OR] 1.04, 95% confidence interval [CI] 1.02–1.06) and cardiovascular diseases (OR 7.02, 95% CI 1.55–31.8) were associated with in-hospital mortality. Platelets less than 150,000/μL (OR 3.14, 95% CI 1.02–9.65) and higher aspartic aminotransferase (OR 1.02, 95% CI 1.00–1.03) were associated with gram-negative rods.

**Conclusions:**

Patients with suspected psoas abscesses or pyogenic spondylitis having a high BUN level and a history of cardiovascular diseases have a higher risk of mortality.

## Background

Pyogenic spondylitis and psoas abscesses are caused by *Staphylococcus aureus*, often in areas with a low prevalence of tuberculosis [1–3]. Patients often have underlying diseases such as malignancies, diabetes mellitus, chronic renal failure, and cirrhosis, as well as long-term corticosteroid use [4–8]. These diseases are diagnosed using a combination of imaging techniques such as computed tomography (CT) or magnetic resonance imaging (MRI) and specimen cultures; however, diagnosis may often be difficult if the patient has few symptoms.

While some studies have reported the underlying diseases associated with pyogenic spondylitis and psoas abscesses [4–8], few have discussed the risk factors for a poor prognosis. It is also important to decide whether to administer antibiotics targeting gram-negative rods because bacteria other than *Staphylococcus* should be considered in some circumstances. Because clinical characteristics and risk factors associated with mortality or bacterial strains have not been well investigated, we described the clinical characteristics of patients with pyogenic spondylitis and psoas abscesses and investigated the factors associated with in-hospital deaths and the presence of gram-negative rods at the time of diagnosis. In addition, we compared the differences in clinical characteristics and outcomes between pyogenic spondylitis and psoas abscess, if any.

## Methods

### Study design and patients

We conducted a historical cohort study of all patients diagnosed with pyogenic spondylitis or psoas abscesses from 2000 to 2014 at Shimane Prefectural Central Hospital, a tertiary care hospital in Japan. Inclusion criteria were (1) patients who were diagnosed with pyogenic spondylitis or psoas abscesses by the physician in charge, (2) confirmation of the clinical diagnosis by radiological images, and (3) no apparent other causes that may mimic pyogenic spondylitis or psoas abscesses. There were no exclusion criteria. The diagnosis of pyogenic spondylitis and psoas abscess was confirmed using either CT or MRI. Bacteria associated with lesions or blood cultures were identified. Surgical interventions, such as percutaneous drainage, surgical drainage, and laminectomy, were determined by the physician in charge. The antimicrobial treatment was determined by the physician in charge based on the culture results and sensitivity analyses. Until the culture results were available or if the causative species could not be determined, empirical treatments based on established guidelines were administered.

We retrieved clinical data, vital signs, and laboratory test results of the patients from the Integrated Intelligent Management System database of Shimane Prefectural Central Hospital between August 1998 and August 2014. The Institutional Review Board of Shimane Prefectural Central Hospital approved this study. Since all data were obtained as part of our routine daily practice, informed consent was waived by the institutional review board.

### Measurements

Clinical data included age, sex, the primary complaint, days from admission to diagnosis of pyogenic spondylitis or psoas abscess, and comorbidities (diabetes, hypertension, hyperlipidemia, cardiac disease, cerebrovascular disease, neurological disease, liver disease, renal disease, malignancy, and surgical history).

We also collected data regarding patient vital signs (systolic blood pressure [SBP], diastolic blood pressure [DBP], heart rate, and body temperature) and laboratory test results (white blood cell count [WBC], hemoglobin, platelet cell count [Plt], C-reactive protein [CRP], aspartic aminotransferase [AST], alanine aminotransferase [ALT], blood sugar, serum albumin [Alb], total bilirubin, lactate dehydrogenase [LDH], blood urea nitrogen [BUN], creatinine [Cr], sodium, and potassium) at the time of diagnosis.

We also collected data regarding treatment modalities (intravenous antimicrobial use and surgical treatments), as well as in-hospital deaths and the length of time in the hospital.

### Statistical analyses

Continuous variables are presented as the mean and standard deviation (SD) or median and interquartile range (IQR), and categorical variables as numbers and percentages. We compared continuous variables with the Student’s *t* test or the Wilcoxon rank-sum test on the basis of the distributions. We compared categorical variables with the *χ*^2^ test when appropriate; otherwise, we used Fisher’s exact test. To explore the factors associated with in-hospital mortality and the presence of gram-negative rods, we constructed multivariate logistic regression models. We analyzed all patients to identify factors associated with in-hospital mortality but selected only culture-positive patients to investigate the factors associated with gram-negative rods.

Included continuous variables were unmodified; however, the units for WBCs and Plts were 100 and 10,000, respectively. For convenience, platelets were only analyzed if less than 150,000/μL. Potential variables were the measured clinical variables described above, and final models were determined after backward selection. Associations are expressed as odds ratio [OR] and 95% confidence intervals [CI]. All statistical analyses were performed using Stata12. All reported *p* values were two-tailed, and *p* values < 0.05 were considered statistically significant.

## Results

### Patient characteristics

A total of 126 patients (72 with pyogenic spondylitis [57%] and 54 with psoas abscesses [43%]) (Table 1) were studied. Their mean age was 72 ± 11 years (range 37–95 years). The number of male patients was 69 (55%). Lumbago or back pain was more frequent in pyogenic spondylitis (49 [68%] vs. 23 [43%], *p* = 0.004), whereas shock was more frequent in psoas abscesses (9 [17%] vs. 2 [2.8%], *p* = 0.009) (Table 1).

**Table 1:** Patients characteristics

	All	Pyogenic spondylitis	Psoas abscess	*p* value
	*n* = 126	*n* = 72	*n* = 54	
Variables	*n* (%) or mean ± SD or median [IQR]			
Male	69 (55)	38 (53)	31 (57)	0.61
Age, year	72 ± 11	74 ± 10	70 ± 11	0.07
Length of stay, days	60 [39–97]	60 [41–106]	58 [36–94]	0.06
In-hospital death	11 (8.7)	3 (4.1)	8 (15)	0.05
Invasive interventions	54 (43)	25 (35)	29 (54)	0.045
Percutaneous drainage	50 (40)	21 (29)	29 (54)	0.006
Operation	6 (4.8)	5 (6.9)	1 (1.9)	0.24
Laminectomy	4 (3.2)	4 (5.6)	0 (0.0)	0.13
Surgical drainage	2 (1.6)	1 (1.4)	1 (1.9)	1.00
Days after admission to diagnosis, day	0 [0–11]	0 [0–5]	2 [0–20]	0.0167
Days after admission to diagnosis, day ≥ 14 days	28 (22)	10 (14)	18 (33)	0.016
Antibiotics use, days	28 [17–42]	28 [18–42]	30 [15–42]	0.79
Over 6 weeks	38 (30)	23 (32)	15 (28)	0.70
Symptoms				
Lumbago or back pain	72 (57)	49 (68)	23 (43)	0.004
Fever	51 (40)	32 (44)	19 (35)	0.30
Shock	11 (8.7)	2 (2.8)	9 (17)	0.009
Classification				
Type A^*^	–	33 (46)	–	–
Type B^*^	–	18 (25)	–	–
Type C^*^	–	21 (29)	–	–
Multiple abscesses	–	–	30 (56)	–
Co-morbidities	102 (81)	56 (78)	46 (85)	0.36
Hospitalized for comorbidity	57 (45)	27 (38)	30 (56)	0.044
Hospitalized for other infections	17 (13)	6 (8.3)	11 (20)	0.07
Bacterial detection	84 (67)	45 (63)	39 (72)	0.34
Gram-positive cocci	63 (50)	35 (49)	28 (52)	0.72
Gram-negative rods	19 (15)	8 (11)	11 (20)	0.15
Mycobacterium	2 (1.6)	2 (2.8)	0 (0.0)	0.51
Vital signs				
SBP, mmHg	132 ± 31	137 ± 28	127 ± 35	0.10
DBP, mmHg	75 ± 18	78 ± 15	70 ± 20	0.0151
Body temperature, °C	37.3 ± 1.1	37.4 ± 1.1	37.2 ± 1.1	0.27
Heart rate, /min	89 ± 19	88 ± 19	89 ± 19	0.30
Laboratory data				
WBC, × 10^2^/μL	113 ± 51	106 ± 41	122 ± 61	0.07
Hb, g/dL	11.3 ± 2.2	11.7 ± 1.8	10.7 ± 2.5	0.0116
Plt, × 10^4^/μL	22.8 ± 11.8	24.8 ± 11.5	20.0 ± 11.9	0.0234
CRP, mg/dL	11.1 ± 9.8	9.4 ± 8.4	13.3 ± 11.0	0.0233
T-bil, mg/dL	0.8 ± 0.5	0.8 ± 0.4	0.8 ± 0.5	0.75
Alb, g/dL	3.3 ± 0.6	3.4 ± 0.6	3.1 ± 0.7	0.0045
AST, IU/L	33 ± 30	31 ± 28	37 ± 31	0.26
ALT, IU/L	26 ± 23	26 ± 25	24 ± 19	0.73
LDH, IU/L	261 ± 123	239 ± 88	290 ± 153	0.0233
Blood sugar, mg/dL	152 ± 71	141 ± 66	165 ± 76	0.06
BUN, mg/dL	25.6 ± 22.8	20.9 ± 10.6	32.0 ± 32.0	0.0069
Cr, mg/dL	1.3 ± 1.7	1.0 ± 0.8	1.7 ± 2.5	0.0178
Na, mmol/L	137.0 ± 5.0	137.2 ± 5.1	136.7 ± 4.8	0.57
K, mmol/L	4.0 ± 0.6	4.0 ± 0.5	3.9 ± 0.7	0.42

*Classification by Pola et al. [17]

All 126 patients received antibiotic treatment. One patient received only oral antibiotics. A total of 54 (43%) patients received invasive interventions, and they were more frequent in psoas abscesses (29 [54%] vs. 25 [35%], *p* = 0.045). The invasive interventions included 50 percutaneous drainage (40%), 4 laminectomy (3.2%), and 2 surgical drainage (1.6%). Two patients received multiple treatments, one patient received percutaneous drainage and laminectomy, another patient received percutaneous drainage and surgical drainage.

There were 11 in-hospital deaths (8.7%). Although there was one death (0.8%) within 14 days and 10 deaths (7.9%) 14 days after admission, these were not statistically significant (*p* = 0.82). When we compared the number of deaths before, and 60 days after admission, there were 6 deaths (4.8%) and 5 deaths (4.0%), respectively.

The number of patients who had comorbidities was 102 (81%), including 36 (29%) with hypertension, 32 (25%) with a surgical history, 21 (17%) with malignancies, 19 (15%) with diabetes, 15 (12%) with neurological diseases, 18 (14%) with cardiac disease, and 15 (12%) with cerebrovascular disease (Table 2).

**Table 2:** Co-morbidities of patients and status at hospitalization

	All	Pyogenic spondylitis	Psoas abscess	*p* value
	*n* = 126	*n* = 72	*n* = 54	
Variables	*n* (%)			
Co-morbidities	102 (81)	56 (78)	46 (85)	0.36
Diabetes	19 (15)	10 (14)	9 (17)	0.80
Hypertension	36 (29)	23 (31)	13 (24)	0.43
Hyperlipidemia	7 (5.6)	5 (6.9)	2 (3.7)	0.70
Cardiac diseases	18 (14)	8 (11)	10 (18)	0.31
Cerebrovascular disease	15 (12)	6 (8.3)	9 (17)	0.17
Neurological disease	15 (12)	8 (11)	7 (13)	0.79
Dementia	4 (3.2)	4 (5.6)	0 (0.0)	0.13
Alcoholism	4 (3.2)	1 (1.4)	3 (5.6)	0.31
Neurosis	2 (1.6)	1 (1.4)	1 (1.9)	1.00
Schizophrenia	2 (1.6)	0 (0.0)	2 (3.7)	0.18
Mental retardation	2 (1.6)	1 (1.4)	1 (1.9)	1.00
Depression	2 (1.6)	1 (1.4)	1 (1.9)	1.00
Epilepsy	1 (0.8)	0 (0.0)	1 (1.9)	0.43
Parkinson’s disease	1 (0.8)	1 (1.4)	0 (0.0)	1.00
Pulmonary disease	7 (5.6)	3 (4.2)	4 (7.4)	0.46
Liver disease	7 (5.6)	4 (5.6)	3 (5.6)	1.00
Renal disease	8 (6.3)	3 (4.2)	5 (9.3)	0.29
Malignancy	21 (17)	9 (13)	12 (22)	0.16
Operation	32 (25)	17 (24)	15 (28)	0.68
Others	30 (24)	17 (24)	13 (24)	1.00
Osteoporosis	5 (4.0)	4 (5.6)	1 (1.9)	0.39
Pancreatitis	2 (1.6)	1 (1.4)	1 (1.9)	1.00
Thyroid disease	3 (2.4)	1 (1.4)	2 (3.7)	0.58
Inguinal hernia	1 (0.8)	0 (0.0)	1 (1.9)	0.43
Malignant syndrome	1 (0.8)	1 (1.4)	0 (0.0)	1.00
Hypoadrenalism	1 (0.8)	1 (1.4)	0 (0.0)	1.00
Gastrointestinal ulcer	4 (3.2)	2 (2.8)	2 (3.7)	1.00
Glaucoma	3 (2.4)	2 (2.8)	1 (1.9)	1.00
Discitis	1 (0.8)	1 (1.4)	0 (0.0)	1.00
Cholecystitis	1 (0.8)	1 (1.4)	0 (0.0)	1.00
Pemphigoid	1 (0.8)	1 (1.4)	0 (0.0)	1.00
Spinal stenosis	1 (0.8)	0 (0.0)	1 (1.9)	0.43
Rheumatoid arthritis	1 (0.8)	0 (0.0)	1 (1.9)	0.43
Reflux esophagitis	2 (1.6)	1 (1.4)	1 (1.9)	1.00
Ureteral stent placement	1 (0.8)	0 (0.0)	1 (1.9)	0.43
Common bile duct stone	1 (0.8)	1 (1.4)	0 (0.0)	1.00
Status at hospitalization				
Hospitalized for comorbidity	57 (45)	27 (38)	30 (56)	0.044
Medical department^*^	40 (32)	19 (26)	21 (39)	0.18
Surgical department^**^	17 (13)	8 (11)	9 (17)	0.43
Hospitalized for other infections	17 (13)	6 (8)	11 (20)	0.07
Urinary tract infection	7 (5.6)	1 (1.4)	6 (11)	0.042
Sepsis	4 (3.2)	2 (2.8)	2 (3.7)	1.00
Pneumonia	2 (1.6)	0 (0.0)	2 (3.7)	0.19
Cholangitis	1 (0.8)	1 (1.4)	0 (0.0)	1.00
Liver abscess	1 (0.8)	0 (0.0)	1 (1.9)	0.43
Pulmonary tuberculosis	1 (0.8)	1 (1.4)	0 (0.0)	1.00
Infectious arthritis	1 (0.8)	1 (1.4)	0 (0.0)	1.00

^*^Comorbidities treated at medical department: blood stream infection 1, cardio-pulmonary arrest 1, cerebral infarction 1, cholangitis 1, congestive heart failure 2, diabetes 1, drug eruption 1, fever of unknown origin 2, gastric ulcer 1, leukemia 1, liver abscess 1, lumbago 2, malignant lymphoma 1, myeloma 2, neuralgia 1, Paget disease 1, pneumonia 3, pulmonary tuberculosis 1, renal failure 3, schizophrenia 1, sepsis 3, skin damage 1, transient ischemic attack 1, urinary tract infection 7

^**^Comorbidities treated at the surgical department: abdominal trauma 1, burn injury 1, colon cancer 2, fall trauma 1, gastric cancer 2, hip pain 1, ileus 3, infectious arthritis 1, internal iliac artery aneurysm 1, knee pain 1, normal pressure hydrocephalus 1, subarachnoid hemorrhage 2

Laboratory testing and physical examinations indicated that CRP (13.3 ± 11.0 vs. 9.4 ± 8.4 mg/dL, *p* = 0.02), LDH (290 ± 153 vs. 239 ± 88 IU/L, *p* = 0.02), BUN (32.0 ± 32.0 vs. 20.9 ± 10.6 mg/dL, *p* = 0.007), and Cr (1.7 ± 2.5 vs. 1.0 ± 0.8 mg/dL, *p* = 0.02) were higher in psoas abscess cases (Table 1).

### Hospital courses

The median time from admission to diagnosis was 0 days (IQR 0–11, minimum 0 and maximum 185). In many cases, hospitalization occurred after the diagnosis of pyogenic spondylitis and psoas abscess (Table 1). The number of patients diagnosed with these diseases ≥ 14 days after hospitalization was 28 (22%) (median 31 days; IQR 21–50, minimum 14 and maximum 185). These patients developed pyogenic spondylitis or psoas abscesses during the course of hospitalization. There were 57 patients who were admitted for other comorbidities: medical department (40 patients) and surgical department (17 patients). Hospitalization for other infections were 17 patients. Comorbidities between pyogenic spondylitis and psoas abscess patients were generally similar (Table 2). Pyogenic spondylitis was diagnosed more rapidly than psoas abscesses (14% in ≥ 14 days vs. 33%, *p* = 0.016). The duration of antibiotics use was a median of 28 days (IQR 17–42, minimum 0 and maximum 206). Thirty-eight patients (30%) received intravenous antibiotics for 6 weeks. There was no statistical difference in the long-term use of antibiotics among patients (*p* = 0.70).

The median length of hospitalization was 60 days (IQR 39–97, minimum 4 and maximum 429). Eleven (8.7%) patients died during the hospitalization period.

Factors associated with in-hospital deaths included a lower SBP (110 ± 35 vs. 134 ± 30 mmHg, *p* = 0.02), a lower DBP (62 ± 19 vs. 76 ± 17 mmHg, *p* = 0.03), lower Alb (2.9 ± 0.8 vs. 3.3 ± 0.6 mg/dL, *p* = 0.02), higher AST (40 ± 19 vs. 33 ± 31 IU/L, *p* = 0.02), higher ALT (29 ± 11 vs. 25 ± 23 IU/L, *p* = 0.04), higher LDH (327 ± 114 vs. 254 ± 122 IU/L, *p* = 0.01), higher BUN (53.5 ± 45.3 vs. 22.9 ± 17.5 mg/dL, *p* = 0.02), and higher Cr (1.7 ± 0.9 vs. 1.3 ± 1.8 mg/dL, *p* = 0.005) (Table 3). The multivariate logistic model showed that BUN (OR 1.04, 95% CI 1.02–1.06) and cardiovascular disease (OR 7.02, 95% CI 1.55–31.8) were associated with in-hospital mortalities (Table 4).

**Table 3:** Factors associated with in-hospital mortality

	Death	Alive	*p* value
	(*n* = 11)	(*n* = 115)	
Variables	*n* (%) or mean ± SD or median [IQR]		
Male	7 (64)	62 (54)	0.75
Age, year	73 ± 10	72 ± 11	0.80
Length of stay, days	57 [34–86]	60 [39–98]	0.68
Diseases			
Psoas abscess	8 (73)	46 (40)	0.038
Invasive interventions	6 (55)	48 (42)	0.31
Percutaneous drainage	6 (55)	44 (38)	0.23
Operation	1 (9.1)	5 (4.3)	0.43
Laminectomy	0 (0.0)	4 (3.5)	1.00
Surgical drainage	1 (9.1)	1 (0.9)	0.17
Co-morbidities	9 (82)	93 (81)	1.00
Diabetes	1 (9.1)	18 (16)	1.00
Hypertension	4 (36)	32 (28)	0.51
Hyperlipidemia	1 (9.1)	6 (5.2)	0.48
Cardiac diseases	4 (36)	14 (12)	0.05
Cerebrovascular disease	1 (9.1)	14 (12)	1.00
Neurological disease	2 (18)	13 (11)	0.62
Pulmonary diseases	0 (0.0)	7 (6.1)	1.00
Liver disease	0 (0.0)	7 (6.1)	1.00
Renal disease	1 (9.1)	7 (6.1)	0.53
Maligancy	2 (18)	19 (17)	1.00
Operation	1 (9.1)	31 (27)	0.29
Others	2 (18)	28 (24)	1.00
Bacteria			
Gram-positive cocci	7 (64)	56 (49)	0.53
Gram-negative rods	0 (0.0)	19 (17)	0.21
Unknown	3 (27)	38 (33)	1.00
Vital signs			
SBP, mmHg	110 ± 35	134 ± 30	0.0196
DBP, mmHg	62 ± 19	76 ± 17	0.0310
Body temperature, °C	36.9 ± 1.1	37.4 ± 1.1	0.12
Heart rate, /min	93 ± 13	89 ± 19	0.33
Labo data			
WBC, × 10^2^/μL	115 ± 56	113 ± 51	0.88
Hb, g/dL	10.2 ± 2.8	11.4 ± 2.1	0.07
Plt, × 10^4^/μL	20.4 ± 18.8	23.0 ± 11.0	0.17
CRP, mg/dL	17.8 ± 12.2	10.4 ± 9.3	0.06
T-bil, mg/dL	1.1 ± 0.7	0.8 ± 0.5	0.11
Alb, g/dL	2.9 ± 0.8	3.3 ± 0.6	0.0220
AST, IU/L	40 ± 19	33 ± 31	0.0245
ALT, IU/L	29 ± 11	25 ± 23	0.0376
LDH, IU/L	327 ± 114	254 ± 122	0.0134
Blood sugar, mg/dL	156 ± 42	151 ± 73	0.26
BUN, mg/dL	53.5 ± 45.3	22.9 ± 17.5	0.0197
Cr, mg/dL	1.7 ± 0.9	1.3 ± 1.8	0.0053
Na, mmol/L	134.6 ± 10.0	137.3 ± 4.2	0.86
K, mmol/L	4.0 ± 0.5	4.0 ± 0.6	0.68

**Table 4:** Multivariate logistic model for death

	Odds ratio	95% confidence interval
BUN, mg/dL	1.04	1.02–1.06
Cardiovascular diseases	7.02	1.55–31.8

### Microbiological examinations

Causal microorganisms were identified in 85 patients (67%), including gram-positive bacteria in 63 patients (50%), gram-negative rods in 19 patients (15%), and others or undetermined (Table 5).

**Table 5:** Causative bacteria

Bacteria	All (*n* = 126)
	*n* (%)
Identified	85 (67)
Gram-positive cocci	63 (50)
Staphylococci	51 (40)
MSSA	28 (22)
MRSA	12 (9.5)
CNS	11 (8.7)
Enterococci	3 (2.4)
Streptococci	8 (6.3)
Gram-negative rods	19 (15)
*Escherichia coli*	7 (5.6)
Klebsiella	3 (2.4)
Prevotella	3 (2.4)
*Proteus mirabilis*	2 (1.6)
*Citrobacter koseri*	1 (0.8)
Bacteroides	4 (3.2)
Mycobacterium	2 (1.6)
*Tuberculosis*	1 (0.8)
Nontuberculosis	1 (0.8)
Other bacteria	2 (1.6)
Unknown	41 (33)

*MSSA* methicillin-sensitive *Staphylococcus aureus*, *MRSA* methicillin-resistant *Staphylococcus aureus*; *CNS* coagulase-negative *Staphylococcus*; *Enterocucci Enterococcus faecium* 1, *Enterococcus faecalis* 2; *Streptcocci* alpha-hemolytic *Streptococcus* 1, *Streptococcus agalactiae* (type B group) 3, *Streptococcus intermedius* 1, *Streptococcus sanguinis* 1, *Streptococcus pneumoniae* 2; *Klebsiella Klebsiella pneumoniae* 2, *Klebsiella oxytoca* 1; *Prevotella Prevotella oris* 1, *Prevotella melaninogenica* 1, unidentified 1; *Bacteroides Bacteroides fragilis* 3, *Bacteroides thetaiotaomicron* 1; other bacteria: Corynebacterium sp. 1

Factors associated with gram-negative rods included lower Plts (15.8 ± 9.6 vs. 22.9 ± 12.3 × 10,000/μL, *p* = 0.0134; Plt < 1.5 × 10^4^/μL, 11 [58%] vs. 20 [30%], *p* = 0.034) and higher ASTs (57 ± 57 vs. 32 ± 23 IU/L, *p* = 0.0236) (Table 6). The multivariate logistic model showed that platelets less than 150,000/μL (OR 3.14, 95% CI 1.02–9.65) and higher aspartic aminotransferase (OR 1.02, 95% CI 1.00–1.03) were associated with gram-negative rods (Table 7).

**Table 6:** Factors associated with gram-negative rods

	GNR	Others	*p* value
	(*n* = 19)	(*n* = 66)	
Variables	*n* (%) or mean ± SD or median [IQR]		
Male	13 (68)	33 (50)	0.20
Age, year	71 ± 9	72 ± 12	0.41
Psoas abscess	11 (58)	29 (44)	0.31
In hospital death	0 (0.0)	8 (12)	0.19
Invasive intervention	11 (58)	35 (53)	0.80
Percutaneous drainage	10 (53)	33 (50)	1.00
Operation	2 (11)	3 (4.5)	0.31
Laminectomy	2 (11)	2 (3.0)	0.22
Surgical drainage	0 (0.0)	1 (1.5)	1.00
Co-morbidities	15 (79)	52 (79)	1.00
Diabetes	3 (16)	9 (14)	0.73
Hypertension	5 (26)	12 (18)	0.52
Hyperlipidemia	2 (11)	3 (4.5)	0.31
Cardiac diseases	4 (21)	9 (14)	0.48
Cerebrovascular disease	5 (26)	8 (12)	0.15
Neurological disease	3 (16)	9 (14)	0.73
Pulmonary disease	0 (0.0)	4 (6.1)	0.57
Liver disease	1 (5.3)	4 (6.1)	1.00
Renal disease	0 (0.0)	4 (6.1)	0.57
Malignancy	1 (5.3)	12 (18)	0.28
Operation	6 (32)	17 (26)	0.77
Others	6 (32)	12 (18)	0.22
Vital signs			
SBP, mmHg	124 ± 27	131 ± 32	0.40
DBP, mmHg	75 ± 18	72 ± 16	0.58
Body tempereture, °C	37.7 ± 1.3	37.4 ± 1.1	0.59
Heart rate, /min	91 ± 17	91 ± 20	0.83
Laboratory			
WBC, ×10^2^/μL	138 ± 69	120 ± 49	0.42
Hb, g/dL	11.8 ± 2.0	11.1 ± 2.1	0.16
Plt, ×10^4^/μL	15.8 ± 9.6	22.9 ± 12.3	0.0134
Plt < 1.5 × 10^4^/μL	11 (58)	20 (30)	0.034
CRP, mg/dL	17.1 ± 9.5	12.6 ± 9.8	0.07
T-bil, mg/dL	1.0 ± 0.6	0.8 ± 0.4	0.18
Alb, g/dL	3.2 ± 0.6	3.2 ± 0.6	0.70
AST, IU/L	57 ± 57	32 ± 23	0.0236
ALT, IU/L	31 ± 23	27 ± 25	0.22
LDH, IU/L	292 ± 126	264 ± 108	0.37
Blood sugar, mg/dL	147 ± 83	157 ± 73	0.23
BUN, mg/dL	26.9 ± 14.9	25.2 ± 20.6	0.19
Cr, mg/dL	1.3 ± 1.2	1.3 ± 1.7	0.24
Na, mmol/L	136.9 ± 3.7	137.0 ± 4.7	0.86
K, mmol/L	4.0 ± 0.6	3.8 ± 0.5	0.66

*GNR* gram-negative rods

**Table 7:** Multivariate logistic model for gram-negative rods

	Odds ratio	95% confidence interval
Plt < 1.5 × 10^4^/μL	3.14	1.02–9.65
AST, IU/L	1.02	1.00–1.03

## Discussion

We showed the epidemiology of pyogenic spondylitis and psoas abscesses, as well as the factors associated with in-hospital mortality and the presence of gram-negative rods in patients’ cultures at a single center. The factors associated with mortality were an elevated BUN and a history of cardiovascular disease. The factors associated with a positive culture of gram-negative rods included higher AST and lower Plt laboratory results.

Previous studies have reported that the predisposing factors for bacterial spondylitis or psoas abscesses were diabetes mellitus, malnutrition, substance abuse, human immunodeficiency virus infection, malignancy, long-term steroid use, chronic renal failure, liver cirrhosis, and sepsis [4–8]. Some reports have showed that CRP or WBCs were associated with recovery [9, 10], although our study showed that CRP was also associated with gram-negative rods.

*Staphylococcus* was found in 50–88% of patients in prior studies [3, 11, 12], and our study showed a similar percentage (60%). Among gram-negative bacteria identified in our study, *Escherichia coli* was found in 5.6%, which was slightly higher than the 2.8% reported in previous studies [11, 13]. *Mycobacterium tuberculosis* is a frequent cause of psoas abscesses in regions where tuberculosis is common (e.g., southern China) [1, 2]; however, the proportion of patients with tuberculosis among pyogenic spondylitis cases decreased to about 24% in these areas [3]. Tuberculosis is common in Japan, yet there was only one case of tuberculosis in our study, which may reflect an early diagnosis before progression to severe tuberculosis or before the incidence of tuberculosis decreased in Japan [14].

In previous studies, delay of treatment, old age, sepsis, and *E. coli* infection were reported as mortality risk factors [11, 15]. There were no differences in mortality between patients with and without gram-negative rods and between elderly and younger patients in our study. We assumed that all patients were promptly treated after the diagnosis. If the treatment was delayed, this factor might be associated with mortality. A previous report revealed an association between endocarditis and pyogenic spondylitis [16]; however, there were no cases of endocarditis in our study. Psoas abscesses are generally reported to have higher morbidity and mortality. One study reported that the mortality rate of primary and secondary abscesses was 2.4% and 19%, respectively, and may approach 100% in untreated cases [1]. Our study had a similar mortality rate (15%), including primary and secondary psoas abscesses, although we could not differentiate them.

When the causative microorganism could not be identified, clinicians must administer an empirical treatment. The empirical treatment policy of the institution was following: (1) vancomycin ± cefazolin in general and (2) meropenem or similar antibiotics when gram-negative bacteria was likely in the setting of previous organism or infections of other sites. Those patients with an elevated BUN or cardiovascular comorbidity were at a higher risk of mortality. Therefore, such patients should receive broad-spectrum antibiotics as well as aggressive drainage and other intensive supportive therapies. The factors associated with gram-negative rods should also be a guide for empirical treatments. The prevalence of gram-negative rods was low, but those with lower platelet counts or elevated ASTs may be at a higher risk of gram-negative rod infections. These patients should receive antibiotics that target gram-negative rods as an initial therapy.

A new classification of pyogenic spondylodiscitis has been reported [17]. The new classification was based on clinical symptoms and radiological findings and associated with recurrence rate and mortality. Since our study had a retrospective design, we could not obtain the information necessary to reclassify our patients and our risk factors should be re-evaluated in future studies incorporating the new classification.

In this study, BUN and a history of cardiovascular disease were associated with in-hospital deaths. Low Plts (< 150,000/μL) and high ASTs were associated with gram-negative rods after performing multivariate analyses. For the group with a higher risk of in-hospital mortality, aggressive drainage should be considered in addition to intensive antimicrobial combination therapy. Although the frequency of gram-negative rods was low, the use of wide-spectrum antibiotics should be considered for the group with a high probability of having gram-negative rods based on these risk factors.

This study has some limitations. First, since our study had a retrospective design, we were unable to measure all factors. Second, we investigated a total of 126 patients, and this sample size is insufficient for robust multivariate analyses. We also could not break down into small homogenous group due to small sample size. However, our primary purpose was to describe the general picture of patients who were diagnosed in daily practice. Third, since we focused solely on patients with pyogenic spondylitis or psoas abscesses and did not analyze all patients who presented with fever and lower back pain, there is a possibility of missed cases. However, considering that our institution is a teaching hospital with easy access to imaging technology, we believe that the number of missed cases is low. Fourth, bacteria were not identified in all cases. Therefore, factors related to gram-negative bacteria should be interpreted with caution. Fifth, there were no established protocols for antibiotics and surgical treatments because this study was a retrospective observational study. The effect of treatment modalities on mortality should be considered. Sixth, we could not classify the psoas abscesses as primary and secondary. If we had been able to differentiate between primary and secondary psoas abscesses, we might have indicated another risk factor for mortality as reported in the previous study. Seventh, there were many variables we compared between pyogenic spondylitis and psoas abscess. The issue of multiple comparisons and the resultant significance should be considered to interpret the results.

## Conclusion

In clinical practice, pyogenic spondylitis and psoas abscesses are likely to be severe in the presence of low blood pressure, malnutrition, liver failure, and kidney dysfunction. When deciding which antibiotic to use, the possibility of gram-negative bacteria should be considered in patients with low Plts and liver dysfunction.

## Abbreviations

Alb: Albumin
ALT: Alanine aminotransferase
AST: Aspartic aminotransferase
BUN: Blood urea nitrogen
CI: Confidence interval
CNS: Coagulase-negative Staphylococcus
Cr: Creatinine
CRP: C-reactive protein
CT: Computed tomography
DBP: Diastolic blood pressure
GNR: Gram-negative rods
IQR: Interquartile range
LDH: Lactate dehydrogenase
MRI: Magnetic resonance imaging
MRSA: Methicillin-resistant Staphylococcus aureus
MSSA: Methicillin-sensitive Staphylococcus aureus
OR: Odds ratio
Plt: Platelet cell count
SBP: Systolic blood pressure
SD: Standard deviation
WBC: White blood cell count
